# Molecular imaging of bacterial infections in vivo: the discrimination of infection from inflammation

**DOI:** 10.3390/informatics1010072

**Published:** 2014-05-30

**Authors:** Heather Eggleston, Peter Panizzi

**Affiliations:** 1 Department of Drug Discovery and Development, Harrison School of Pharmacy, Auburn University, Auburn, AL 36849

**Keywords:** Molecular Imaging, Endocarditis, Infection, Inflammation, Staphylococcus aureus

## Abstract

Molecular imaging by definition is the visualization of molecular and cellular processes within a given system. The modalities and reagents described here represent a diverse array spanning both pre-clinical and clinical applications. Innovations in probe design and technologies would greatly benefit therapeutic outcomes by enhancing diagnostic accuracy and assessment of acute therapy. Opportunistic pathogens continue to pose a worldwide threat, despite advancements in treatment strategies, which highlights the continued need for improved diagnostics. In this review, we present a summary of the current clinical protocol for the imaging of a suspected infection, methods currently in development to optimize this imaging process, and finally, insight into endocarditis as a model of infectious disease in immediate need of improved diagnostic methods.

## 1. Clinical Approach to Identification of Infection

Identification of generalized infection with imaging modalities relies upon monitoring morphological changes with radiograph, ultrasonography, computed tomography (CT), and magnetic resonance imaging (MRI). CT and MRI provide particularly useful data in detecting organ and musculoskeletal infections; however, the data collected from these imaging modalities is often only attainable in late stages of infection and is further complicated by morphological distortion induced by post-surgical changes, scarring, and presence of foreign materials [1]. Most imaging modalities are further limited by their inability to distinguish (i) inflammation from infection, (ii) tumors from abscesses, and (iii) causative pathogens. There is a rich history of the use of radiolabeled markers (i.e. proteins and cells) for imaging infectious processes by either single-photon emission computed tomography (SPECT) or positron emission tomography (PET) as a complement to these aforementioned techniques for morphological imaging. Examples of radiolabelling isotopes that are most common include ^99m^Technetium (^99m^Tc), ^111^Indium (^111^In), ^68^Gallium salts (^68^Ga), and ^18^Fluorine (^18^F), which have been applied to labeling leukocytes and their cellular products, in addition to labeling therapeutic molecules such as antibiotics, monoclonal antibodies, and experimental therapeutics by use of chelator such as diethylene triamine penta-acetic acid (DPTA), 1,4,7,10-tetraazacyclododecane-1, 4, 7, 10-tetraacetic acid (DOTA), and hexamethylproplyleneamine oxime (HMPAO) [2]. The use of such chelators allows for consideration of multiple isotopes due to their ability to modulate the imaging window by compensating for differences in the half-life and function of the isotope (i.e. gamma emission). A summary of the currently available labeling methods and specific details related to their use can be found in Table 1. Other examples require the accumulation of patient derived leukocytes, labeled with ^111^Inoxine or ^99m^Tc-HMPAO and re-injected into the donor patient, for the clinical detection of an underlying infection. The current clinical method utilizing labeled leukocytes is recommended for a range of inflammatory disorders and infections; the differentiation between sites of sterile inflammation and infection relies upon optimizing image acquisition and interpretation at predetermined time points [3, 4, 5]. The reliance upon the specificity of image acquisition and interpretation as opposed to the specificity of the reagent highlights the need for pathogen-specific probes as opposed to infection-associated inflammation.

## 2. Separating Inflammation from Infection

Discrimination of generalized inflammation from infection is not easily obtained, primarily due to similarities in immune response generated by tissue damage or chronic insult. As a result, currently employed imaging techniques rely largely on the detection of inflammation associated with infection. However, the potential inaccuracy of this assumption limits the efficacy of this approach, and necessitates additional confirmation of the underlying infection, by positive blood tests or biopsies and non-specific symptoms of the patients, such as fever and general malaise. It is important to note, however, that the identification of immune cells, their receptors, and products, which exhibit up-regulation or increased specificity in the infectious process may be utilized as molecular targets for the monitoring of inflammation associated with infection. Host responses to infectious stimuli trigger overlapping responses that include an initial release of histamines with concurrent elaboration of inflammatory cytokines, followed by a rapid neutrophil burst response to these triggers, prolonged splenic and tissue release of monocytes to the site of damage, tissue conversion of monocytes to macrophages to aid in engulfment and lysis of the foreign pathogens, and later followed by lymphoid generation of pathogen specific T cells and high affinity B cell antibodies. Therefore, we have outlined here a current summary of methods specifically used to detect these immune cell types and their distinguishing products.

### 2.1. Indirect Detection of Leukocytes

#### 2.1.1. Integrins and Selectins

Although current clinical standards involve the *ex vivo* labeling of patient derived leukocytes, non-invasive methods have been developed to indirectly detect cells through the up regulation of selectin and integrin leukocyte receptors during inflammatory processes. Vascular cell adhesion molecule-1 (VCAM-1) expression is highly up-regulated on endothelial cells as a response to inflammatory cytokines to promote the adhesion of leukocytes, particularly slowing cells rolling from the vasculature, by binding to very late antigen 4 (VLA-4) and subsequent participation in leukocyte-endothelial signal communication. VLA-4 conjugated to VCAM-1 encapsulated in a cross-linked iron oxide nanoparticles (CLIO) has been show to detect the VCAM-1 expression associated with atherosclerotic plaques [6, 7, 8]. A molecule similar to VCAM-1, Intercellular adhesion molecule 1 (ICAM-1) is displayed by the activated endothelium, macrophages, and lymphocytes upon exposure to the cytokines Interluekin-1 (IL-1) and tumor necrosis factor-α (TNF-α), and allows for the transmigration of leukocytes through the endothelium. To detect relative ICAM-1 levels by MRI, Wong *et al*. developed a superparamagnetic iron oxide (SPIO)-based nanomicelle coated with lymphocyte function-associated antigen 1 (LFA-1) that binds specifically to ICAM-1 and Choi *et al*. developed a Gd-DPTA-anti-ICAM-1 antibody [9, 10].

P-selectin and E-selectin are integrins that are commonly upregulated as a result of inflammation. Imaging of P-selectin has been achieved by several methods: ^99m^ Tc-labeled, ^111^In-labeled-, and Cy7-labeled-anti-P-selectin monoclonal antibody; fucoidan, a ligand of P-selectin with an affinity in the nanomolar range, has been labeled with ^99m^Tc; FITC labeled monoclonal antibody, anti-humanCD62P (P-selectin); the development of versatile ultra-small paramagnetic iron oxide nanoparticles (VUSPIO) consisting of PEG and dextran coated iron oxide nanoparticles conjugated with an anti-human-P-selectin monoclonal antibody for MRI; and microparticles of iron oxide with dual ligands of VCAM-1 and P-selectin, also for MRI [11, 12, 13, 14, 15]. ^111^-In-labeled and ^99m^Tc-labeled monoclonal antibodies of E-selectin allow for detection of E-selectin positive immune cells in the inflammatory microenvironment; a comparison of the two methods in a clinical trial of 10 patients with rheumatoid arthritis demonstrated ^111^In-labeled and ^99m^Tc-labeled anti-E-selectin monoclonal antibody have equivalent efficacy in the detection of active inflammation within joints, but ^99m^Tc is a more readily available radioisotope with a preferred imaging time of four hours [16, 17, 18]. A Gd- DPTA nanoparticle with a Sialyl-Lewis^x^ motif that binds E-selectin was shown to localize to endothelial activation within the brain [19].

#### 2.1.2. Myeloperoxidase

Reporters directed at products elaborated by these immune cells can also be target to assess inflammation that may exist as a result of underlying infection. A central enzyme in inflammatory immune response, myeloperoxidase (MPO) is produced by myeloid cells and generates reactive species, such as hypochlorous acid and oxygen radicals that damage tissues and pathogens. Several agents have been developed that detect MPO and its byproducts; for example, standard hydrogen peroxidase or hydrogen peroxide sensing reagents can be used *in vivo* for this goal. This is typified by the use of luminol as a chemiluminescent light reporter by two MPO dependent mechanisms: the luminol reacts with a radical oxygen produced by NADPH oxidase, and is subsequently oxidized by MPO, or it reacts with the hypochlorous acid produced by the reaction of MPO with hydrogen peroxide; each reaction results in the chemiluminescent molecule 3-aminophthalate [22]. A comparable substrate, pholasin, a glycoprotein that reacts with reactive oxygen species (ROS), may be superior to luminol in its method of action due to its increased sensitivity and accelerated degradation [23]. Utilizing two substrates, (DOTA)-Gd and bis-5-HT-DOTA-Gd, that form radicals and oligomers in the presence of MPO, MPO can be detected by MRI as an increase in the relaxivity of the tissue [24]. Sulfonaphthoaminophenyl fluorescein (SNAPF) is a fluorescein probe that responds to the hypochlorous acid produced when MPO catalyzes the oxidation of hydrogen peroxide in the presence of chloride ions in murine and human tissue [25]. Non-specific fluorescein based probes developed for ROS detection include: a napthofluorescein-based near-infrared fluorescent probe, Naphtho-Peroxyfluor-1 (NPF1), which indicates hydrogen peroxide levels within macrophages as measured by flow cytometry [26]; 2-[6-(4_-hydroxy)phenoxy-3H-xanthen-3-on-9-yl]benzoic acid (HPF) and 2-[6-(4-amino)phenoxy-3H-xanthen-3-on-9-yl]benzoic acid (APF) auto-oxidation resistant probes which produce fluorescein upon reaction with specific ROS, and in combination, can discriminate between highly reactive oxygen species and hypochlorite [27]. 5-(and-6)-chloromethyl-2′,7′-dichlorodihydrofluorescein diacetate (CM-H2DCFDA) is a reduced fluorescein probe that permeates the cell, reacts with intracellular ROS, and is retained within the cell (LifeTechnologies).

Potential clinically applicable ROS sensitive probes include antioxidant nanoparticles that degrade into non-toxic and anti-inflammatory components upon exposure to hydrogen peroxide, and then inhibit the generation of ROS by *in vivo* macrophages [28], and a biocompatible nanoparticle coated with 400 quenched oxazine molecules, which are activated upon interaction with peroxynitrite and hypochlorous acid produced by MPO [29]. The advantage of imaging MPO reaction products based on the nanoparticle scaffold is that the nanoprobe has a half-life conducive to *in vivo* imaging. In development of the probe, we tested the ability of the MPO sensor to signal inflammatory response in a myocardial infraction model based on permanent ligation of the descending coronary artery. The MPO sensor was given via tail-vein injection at the height of the myeloid inflammatory response and, as the monocytes and neutrophils were recruited to the damaged myocardial, the probe was oxidized by peroxynitrite and hypochlorous acid generated in the cells and released into the environment (i.e. oxazine was liberated from the MPO sensor). Although only tested by flow cytometry using neutrophils isolated from splenocytes, this MPO sensor has the ability to respond to hydrazine-based inhibition and may be of use in the evaluation of the *in vivo* efficacy of MPO-based cleavage and heme liberation caused by various hydrazine analogs [21]. MPO is an excellent inflammatory target but would have no ability to discriminate types of pathogens.

### 2.2. Detection of Myeloid Cells

#### 2.2.1. Monocytes and Macrophages

The differentiation of monocytes to tissue macrophages occurs in the presence of tissue damage or pathogens. Tissue macrophages phagocytose pathogens and apoptotic cells and generate signaling molecules to recruit additional immune cells [30]. Their phagocytic function enables the absorption of iron oxide nanoparticles (CLIO, SPIO, USPIO) [31] and ^19^F-labeled-perfluorotributylamine (PFTA) coated particles for MR imaging [32]; additional labeling methods include ^89^-Zr-dextran coated nanoparticles (DNP) [33] and ^64^Cu-DTPA-monocrystalline iron oxide nanoparticles (MION) [34], both of which are visualized by hybrid PET/MRI; Macrophage scavenger receptor (MSR) targeted-Gd containing immunomicelles [35] and Gd containing lipid based nanoparticles targeted for the macrophage scavenger receptor-B (CD36) for MR imaging with enhanced macrophage specificity [31, 36]. In a comparison study of sterile inflammation and osteomyelitis, injection of USPIO and subsequent macrophage uptake resulted in USPIO-enhanced macrophage localization in infectious vertebral osteomyelitis as opposed to limited macrophage infiltration in sterile vertebral inflammation [37].

#### 2.2.2. Neutrophils

Neutrophils function as key mediators of inflammation and infection due to their phagocytic function and production of ROS in a process termed respiratory burst. Several of the neutrophil specific agents developed utilize the PET agent ^99m^Tc: ^99m^Tc-hydrazinonicotinic acid (HYNIC)-Neutrophil activating peptide-2 (NAP-2) [38]; ^99m^Tc-IL-8 is a chemotactic cytokine secreted by macrophages which binds with strong affinity to receptors on neutrophils [39]; ^99m^Tc-antiCD15-IgM monoclonal antibody (LeuTech) binds specifically to both circulating and sequestered neutrophils [40]; leukotriene B-4 (LTB4), a potent chemoattractant of neutrophils, targeted by ^99m^Tc-labeled or ^18^F-HYNIC-labeled LTB4 antagonist [41]. In addition to these ^99m^Tc-labeled targets, two probes have been developed for the formyl peptide receptor displayed by neutrophils: one contains cyanine7 (Cy7) dye conjugated to the formyl peptide mimetic, termed Cy7-PEG-cFIFIFK for pre-clinical fluorescence imaging [42], as well as a cFLFLFK-PEG-^64^Cu-DOTA for MRI [43]. Neutrophils exhibit increased rates of metabolic activity during active infection, and therefore exhibit a high uptake of ^18^F-FDG [44], ^68^Gallium salts [45], and indocyanine green (ICG) [46]. In addition, the previously mentioned applications of MPO may also be applied to neutrophils, as MPO constitutes the majority (5%) of their azurophilic granules.

### 2.3. Adaptive Immunity

The tracking of adaptive immune cells is not a new idea for imaging infection and inflammatory diseases; such studies include appreciation of *in vivo* dendritic cells [47], T cells [48], and B content [49], with recent advancements in the development of contrast agents for MRI [50].

#### 2.3.1. Dendritic Cells

Dendritic cells are professional antigen presenting cells that are present at the initiation of sites of infection and inflammation, and then migrate to the lymph nodes and spleen to stimulate the differentiation of B and T cells. Methods developed for the labeling of dendritic cells include: perfluoropolyether labeled dendritic cells that can be detected of ^19^F by MRI [51]; a combination of furoxamide and ^111^In-labeled dendritic cells monitored by a combination of SPECT and MRI [52]; these methods have been developed for the noninvasive, long term monitoring of cellular therapy [53, 54, 55].

#### 2.3.2. T cells

T cells are responsible for pathogen immunity by cytolysis of infected cells (CD8+) and activation of B cell affinity maturation (CD4+). Interleukin-2 (IL-2), produced by T cells, stimulates the proliferation of T cells into CD4+ and CD8+ cells; therefore, the detection of IL-2 denotes a pro-inflammatory environment as is consistent with acute infection and chronic inflammatory diseases. To this end, currently 3 PET agents have been developed: ^99m^Tc-labeled IL-2 [56, 57], ^18^F-lableled-IL-2 [58], ^35^S-labeled IL-2, and ^123 or 131^-In-labeled IL-2 [59, 60] to detect human activated T lymphocytes [61]. Two CD8+ T cell specific imaging agents have been developed; they each consist of “mini antibodies,” which are derived from parental antibodies specific to primary CD8+ T cells in the peripheral blood, spleen, and lymph nodes. The engineering of mAbs prevents the depletion of CD8+ T cells *in vivo*; these mAbs are then conjugated to S-2-(4-isothiocyanatobenzyl)-1,4,7-triazacyclononane-1,4,7-triacetic acid for ^64^Cu radiolabeling for immuno-PET imaging [62]. T cells and B cells are difficult to label with iron oxide nanoparticles, therefore manganese chloride, a contrast agent that enhances *T_1_* relaxivity, may be utilized instead [63]. However, the internalization of the paramagnetic agent decreases the *T_1_* relaxivity, reducing the detection strength by MRI.

#### 2.3.3. B cells

Primary B cell labeling involves the labeling of their cell specific products, antibodies. Monoclonal antibodies have undergone optimization of the radionuclide, the chelating agent, and the antibody construct due to their dual diagnostic and therapeutic ability [64]. In addition to directly labeling antibodies, antibody pre-targeting has been developed. This method consists of an injection of an unlabeled artificial antibody conjugate which binds to a specific antibody, accumulates in the solid tumor (or other area of interest), and then is subsequently imaged by the addition of a high avidity effector molecule that binds to the antibody-conjugate pair. Although this method is currently optimized for oncologic application, the method has relevance to similar infectious disease processes, such as abscesses or other areas of localized infection [65]. Direct labeling of the B cells has been attempted with SPIO nanoparticles in conjugation with NIRF dyes for monitoring B cell dynamics within the spleen. However, the introduction of SPIO nanoparticles appeared to interfere with B cell function, therefore labeling methods require further optimization [66]. In general, the attempts to label a multiplicity of cell types, including B cells, T cells, and dendritic cells are limited to labeling with pre-clinical probes, such as NIRF, GFP, and quantum dots for detection by intravital microscopy or flow cytometry [49]. These methods, though valuable in pre-clinical experimentation, do not provide a promise of translation to clinical application.

### 2.4. Small Molecule Imaging

#### 2.4.1. Chemokines

Chemokines are a subset of chemotactic cytokines that are secreted at the site of infection or inflammation for the recruitment of immune cells, and therefore have been explored as imaging targets for detection of cell specific immunity [67]. Notably, monocyte chemoattractant protein-1 (MCP-1) has been labeled with ^125^In allowing for imaging of inflammatory processes which involve monocytes as the primary mediators of inflammation [68]. Another ^125^In-labeled chemokine, platelet factor-4 (PF-4) synergizes with the interleukin-8 (IL-8) that binds with strong affinity to neutrophil receptors, therefore allowing the visualization of neutrophils in inflammatory processes [69].

#### 2.4.2. Proteases

Imaging protease activity has key benefits to tracking inflammation and infectious disease. Arguably there are three families of proteases that are most often associated: matrix-metalloproteinase (MMP), cathepsins, and caspases. MMPs are zinc dependent proteases capable of degrading extracellular matrices, activating and inactivating chemokines and cytokines, and cleaving ligands and cell surface receptors during cell proliferation, angiogenesis, apoptosis, and cell migration [70]. Broadly, the MMPs family members are classified as collagenase (MMP-1, −8, −13, and −18), gelatinase (MMP-2 and −9), stromelysin (MMP-3, −10, and −11), and matrilysin (MMP-7 and −26). To detect these various MMP subtypes, various probes have been developed: a highly lipophilic ^111^In or ^99m^Tc-DTPA-Cys-Thr-Thr-His-Trp-Gly-Phe-Thr-leu-Cys-OH (^111^In or ^99m^Tc-DTPA-CTT) used to image and inhibit MMP-2 preferentially [71]; a conjugation of an MMP-2 substrate with a quenched fluorophore released upon substrate recognition and cleavage [72]; a near infrared polymer-based proteolytic beacon “PB-M7NIR,” consisting of a pegylated dendrimer core covalently coupled to a Cy5.5 labeled MMP-7 specific peptide substrate, which preferentially fluoresces in *in vivo* MMP-7 positive tumors relative to a bilateral control tumor [73]. Bremer *et al.* developed a high-density probe conjugated polymer containing a MMP-2 specific substrate that is quenched prior to MMP-2 mediated protease release [74]; PerkinElmer now provides MMPsense, a probe with a broad range of fluorescent labels (AlexaFluor680, 750, etc.) and MMP specificity (MMP-2, 3, 7, 9, 12, and 13). A similar method was employed by the group in the development of the Prosense reporter, a pan-cathepsin probe for the detection of cathepsin B, H, or L: PCG (protected graft co-polymer) is conjugated to Cy5.5, which allows the cathepsin to cleave the poly-L-lysine backbone of PCG, releasing the quenched fluorophores of PCG and Cy5.5 [75]. This identical method has been applied to MMPs, caspase-1, cathepsin D, and urokinase plasminogen activator as well.

#### 2.4.3. Caspases

In addition to their involvement in apoptosis, specific caspases, such as caspase-1, 3, and 8 have been identified as activated or inhibited in bacterial and viral infections [76, 77, 78]. Targeted photodynamic therapy induces the apoptosis cascade via caspase-9, caspase-8, and caspase-3, and when combined with a caspase-3 activated fluorescent substrate allows for the monitoring of therapeutics [79, 80, 81]. Weissleder *et al.* has developed a biocompatible NIR probe which is (ICE)-specific to cleavage by caspase-1 and whose activity has been demonstrated with whole body NIRF imaging [82]. Each of these proteases is united in a common process, apoptosis, and therefore determining their activity provides additional knowledge about the initiation, progression, or cessation of cell death. The probes specifically developed for the detection of apoptotic cells focus on the abnormal cell morphology characteristic of these cells. For example, ^99m^Tc-labeled bis(zinc(II)-dipicolylamine) (Zn-DPA), a mimetic of annexin V, and annexin V, labeled with one of a variety of reporters including Cy5.5, Gd-DPTA-quantum dot, ^18^F, fluorescein isothiocyanate (FITC), or ^99m^Tc-HYNIC, specifically bind to the phosphatidyl serine exposed on the surface of an apoptotic cell, allowing for the detection of apoptotic cells with multiple imaging modalities [83, 84, 85, 86, 87, 88]. Additionally, ^131^Iodine labeled peptides are caspase substrates absorbed by apoptotic cells [89].

## 3. Imaging of Infectious Species

To increase the ease of pre-clinical experimentation many species of bacteria have been engineered to express a version of the luciferase enzymatic system for the generation of bioluminescence in pre-clinical studies of gene regulation and antibiotic efficacy [90, 91]. Standard protocol for clinical imaging of infection utilizes exogenously radiolabeled patient derived leukocytes, in addition to ^99m^ Tc, ^67^Ga, and ^18^F-FDG tracers for PET imaging due to their absorbance by cells exhibiting high metabolic rates; each method therefore localizes to sites of active bacterial infection with increased extravasation and diapedesis of leukocytes. However, these methods mentioned previously cannot differentiate between infection and inflammation, and therefore cannot separate post-operative inflammation or infection, a critical diagnostic difference for therapeutic efficacy.

### 3.1. Labeled Antimicrobials for Detection of Infection

#### 3.1.1. Synthetic and Endogenous Antibiotics

Labeled antibiotics present a promising method as they localize specifically to the site of an infection, and depending upon their target, are able to identify specific microorganisms. Fluoroquinolones are a class of antibiotics known to intercalate into the DNA of most bacterial species, and therefore have been labeled with ^99m^Tc and ^18^F for PET imaging [92, 93, 94]. Infecton©, a ^99m^Tc-labeled version of Ciprofloxacin, is a clinically approved agent that has been shown to have equivalent or greater efficacy in the detection of musculoskeletal bacterial infections as other clinical agents such as ^18^F-FDG and radiolabeled leukocytes. However, it is noted that Infecton© was removed from the market due to disagreement about the specificity of diagnosis due to incongruity of differentiation between sterile inflammation and infection at multiple time points [92, 93, 94, 95, 96, 97, 98, 99, 100, 101]. A specific diagnostic agent for the detection of Gram-positive bacteria utilizes magnetic nanoparticles derivatized with vancomycin to form CLIO-vanco nanoparticles. This method relies on the avidity of the pathogen binding to 10-100's of these iron oxide core sensors resulting in a *T1*-relaxivity change signaling the presence of Gram-positive bacteria. These nanoparticles have been specifically shown to identify the Gram-positive bacterium *Staphylococcus aureus* [102, 103]. Naturally occurring antibiotic mechanisms, such as antimicrobial peptides and bacteriophages, have also been exploited for imaging by labeling with ^99m^Tc: this includes the non-specific antimicrobial peptides lactoferrin, defensins, ubiquicidin, and human neutrophil peptide-1 (an α-defensin) and the M13 bacteriophage, which exhibited specificity for bacterial strains of *Escherichia coli* and *Staphylococcus aureus*, and when administered, reduced levels of live *E. coli* in a mouse thigh infection model [104, 105, 106, 107, 108, 109, 110]. The high degree of specificity for an intended target and dual diagnostic and therapeutic ability of the ^99m^Tc-labeled bacteriophage has encouraged the expansion to investigation other phage types [111]. There is a current controversy about the merits and demerits of radiolabeled synthetic antibiotics and endogenous antimicrobial peptides. Briefly, fluoroquinolone antibiotics currently optimized for imaging exhibit a non-preferred accumulation in sterile inflammatory sites, in addition to the concerns about the increasing rise of antibiotic resistance that may generate a false negative diagnosis; however, this method has exhibited a higher degree of specificity than ex vivo radiolabeled leukocytes and does not accumulate in the bone marrow, which are important distinctions in the detection of infections such as osteomyelitis, septic arthritis, and infection of orthopedic prostheses. Endogenous antimicrobial peptides exhibit comparable specificity and accuracy to fluoroquinolone antibiotics in the detection of extracellular bacteria, but exhibit none to minimal accumulation in sites of sterile inflammation; however, the greatest concern lies in the development of resistance and subsequent loss of the innate protective mechanism of antimicrobial peptides [112]. A promising antimicrobial peptide, UBI29-41, is a clinically tested agent derived from ubiquicidin, a defensin isolated from human airway epithelial cells. UBI29-41, labeled with ^99m^Tc, was rigorously tested in animal models, and when translated into Phase I clinical trials showed overall sensitivity, specificity, and accuracy of 100%, 80%, and 94.4%, respectively, in patients with soft tissue infections and osteomyelitis with an optimum time for imaging being 30 minutes after intravenous administration of the radiotracer. It was also determined that the detection of the radiotracer was dependent on the number of viable bacteria present, as determined after serial treatment with ciprofloxacin; this can be considered as an advantage in determining the efficacy of antibiotic treatment, but a disadvantage in detecting chronic infections with lower numbers of bacteria that may also be encased within biofilms [112, 113, 114, 115]. A study conducted compared the specificity of ^99m^Tc labeled synthetic antimicrobial peptides (UBI 29-41, 18-35, 31-38 and hLf 1-11), human neutrophil peptides (defensins), and ^99m^Tc-ciprofloxacin (Infecton) in differentiating sites of sterile inflammation and infection [106]. Infection was initiated by injection of multi-drug resistant Gram-positive bacteria (*S. aureus*), Gram-negative bacteria (*Klebsiella pneumonia*), or flucanzole resistant fungi (*Candida albicans*), while sterile inflammation was induced by injection of heat killed microorganisms or lipopolysaccharide (LPS). Results of this study indicated that antimicrobial peptides accumulate specifically in sites of infection; this is proposed to be due to the preferential binding of these peptides to live microorganisms, not activated host leukocytes. ^99m^Tc-ciprofloxacin accumulated in sites of sterile inflammation and infection, therefore the authors concluded that ^99m^Tc-UBI peptides exhibit preferable discrimination of infection from inflammation. This study reinforces the variable ability of ^99m^Tc-ciprofloxacin to discriminate sterile inflammation from infection; ^99m^Tc-ciprofloxacin has demonstrated interactions with mammalian cells, including mammalian DNA, DNA gyrase, topoisomerase II, and human leukocytes and endothelial cells, which contributes to the lack of specificity. In addition, the increasing prevalence of drug resistant microorganisms that either subvert the therapeutic mechanism or efflux the molecule limits the binding of labeled drug molecules [116, 117, 118, 119, 120, 121, 122].

### 3.2. Pathogen Specific Targets

In order to optimize the accuracy of imaging clinical infections, targets should include components unique to the infectious species, preferably with species specificity, or specific to the host immune response to infection. Identifying components of the bacterium include the cell wall, bacterial specific enzymes, and specific host factors acquired for growth.

#### 3.2.1 Cell Wall

The unique composition of the bacterial cell wall allows for the development of probes with high specificity. Wheat germ agglutinin, a lectin, conjugated to colloidal quantum dots allows for specific binding to the N-acetylglucosamine and sialic acid of Gram-positive, not Gram-negative, bacterial cell walls [123, 124]. Non-specific probes which label both Gram-positive and Gram-negative cell walls include fluorescently labeled D-isomer amino acids which are incorporated into newly synthesized peptidoglycan of bacterial cell walls [125]; ^111^In-Zn-DPTA and Cyanine-Zn-DPTA have both been shown to bind to bacterial cell walls, for PET and fluorescence imaging respectively [126, 127, 128]. In addition, Perkin Elmer has developed Xenolight Rediject bacterial detection probe, a pre-clinical NIRF probe targeted for anionic phospholipids that binds with higher affinity to Gram-negative cell walls, but binds at a comparable concentration to Gram-positive cell walls. For a species-specific diagnostic, SPIO nanoparticles conjugated to an antibody for the cell wall of *Mycobacterium tuberculosis* detect extra pulmonary *M. tuberculosis* infection [129].

#### 3.2.2 Bacterial Specific Factors

Specific co-factors required for bacterial growth have also been exploited for pre-clinical imaging, including targets such as biotin and iron. ^111^-In-DOTA-biotin and zinc-dipicolylamine analog (Zn-DPA)-biotin, non-covalently linked by streptavidin (SA) to form the complex ^111^In-DOTA-biotin-SA-Zn-DPA-biotin, has been developed for enhanced visualization upon bacterial absorption with SPECT-CT imaging [126]. Iron must be seized from the host environment; therefore quantum dots with human transferrin conjugates are internalized by, and therefore label the bacterium. However, these quantum dots have been shown to increase the survival of *S. aureus* in iron poor environments, and therefore are not applicable in pre-clinical applications [124]. Bacteria also express a thymidine kinase that differs from human thymidine kinase. The radiolabel, ^124^I-FIAU, is a substrate for the thymidine kinase of bacteria and therefore can be used as an agent to identify musculoskeletal infections by PET-CT [130]. A *S. aureus* specific probe composed of synthetic oligonucleotides flanked by a fluorophore and quencher molecule, termed the Cy5.5-TT probe, was activated upon interaction with the micrococcal nuclease secreted by *S. aureus* [131]. Labeled iron oxide or gold nanoparticles may be absorbed by the bacterium and therefore imaged with MRI, although it is necessary to note that macrophages phagocytosis these particles as well, decreasing the specificity of the diagnosis [132].

## 4. Endocarditis: A Model of Difficult Diagnosis

Endocarditis is an infection of the heart valve and early detection typifies the need for advancements to promote early diagnosis and assessment of causative microorganism [133]. Initial damage to the heart valve denudes the protective cardiac endothelium leading to a sterile clot, which consists of platelets, coagulation factors, fibrin and, in some areas, basement collagen and stroma. These initial sites of damage are often referred to as “sterile” vegetations. Concurrently, bacteremia by opportunistic pathogens such as *S. aureus* can lead to the formation of bacterial vegetations that weaken the valve, leading to regurgitation and ultimately, heart failure.

Treatment options for patients diagnosed with endocarditis rely heavy on aggressive antibiotic therapy, often lasting up to 4-6 weeks. Although removal of the infected valve may prove necessary, this surgical intervention is complicated by the aforementioned difficulty of diagnosis. Therefore, more discriminatory imaging methods will greatly improve therapeutic efficacy and reduce patient morality. Guidelines for diagnosis of endocarditis rely on the modified Duke criteria including (i) a fever, (ii) a new heart mummer, (iii) a positive blood culture for *Staphylococcus aureus*, *Streptococcus* species typical of IE, including viridans streptococci and *Streptococcus bovis*, or other microorganisms from persistently positive blood cultures consistent with IE and (iv) a positive transthoracic or transesophageal echocardiogram (TEE or TTE, respectively) [134, 135]. Often serial TEE and TTE are required to determine if there is growth of the fibrin-bacterial-platelet vegetations. These echo-based methods depend on interpretation by a trained Radiologist, and do not inform on the causative pathogen [136, 137, 138]. Therefore, confirmative PET, SPECT, and MRI agents have been developed to complement this traditional method. Table 2 contains a summary of both clinical and preclinical probes that have been studied for the detection of endocarditis along with useful parameters for discriminating the merit of these findings. The clinical PET agent ^18^F-FDG has been shown to identify cardiac vegetations, particularly in cases of prosthetic valve endocarditis (PVE), though high uptake of 18F-FDG in the physiologically normal myocardium remains a concern [139, 140, 141, 142]. MRI provides anatomical and functional imaging that allows for the detection of perivalvular abscesses and differentiation of pseudoaneurysms from infective endocarditis [143]. The majority of the newly developed techniques utilize the SPECT/CT imaging modality. The clinical agent ^99m^Tc has been used as a label for HMPAO-WBC (white blood cells), anti-NCA-95, an anti-granulocyte antibody for immunoscintigraphy, stannous pyrophosphate in combination with cardiac scintigraphy, and Annexin V for detection by scintigraphy of platelet activation in experimental endocarditis; ^111^Indium has been utilized as a label for platelets and leukocytes with varying success in sensitivity [144, 145, 146, 147, 148]. Pre-clinical probes that could be applied for identification of bacteria within the vegetation of IE include synthetic complexes that target the anionic bacterial cell wall [149, 150]. The targeting of fibrin within the vegetation, in either non-infective or infective endocarditis, would aid in the detection of lesions missed during serial echocardiography. To this aim, Gd-DPTA nanoparticles coated with anti-fibrin monoclonal antibody were developed and tested in an *in vivo* canine thrombus model; it has also been shown that fibrin targeted antibodies labeled with either ^111^In or ^99m^Tc detected and inhibited the vegetative growth of *Streptococcus sanguinis*. Monoclonal antibodies for fibrin termed GC4 and T2G1, developed by Rosebrough et.al, and D59A, developed by Hui et.al, labeled with^131^Iodine or ^111^Indium have been tested in pre-clinical animal studies; D59A has been clinically evaluated for detection for deep vein thrombosis (DVT) (Table 2). These antibodies has been shown to be specific for venous thrombi, due in part to the lack of cross-reactivity with fibrinogen [151, 152, 153, 154, 155, 156, 157, 158, 159]. A clinically approved agent, Thromboview©, is a ^99m^-Tc labeled humanized monoclonal antibody for the D dimer of cross-linked fibrin utilized for the detection of DVT and pulmonary emboli [160, 161]. Two pre-clinical probes specific to coagulase positive *S. aureus*, a major causal agent of infective endocarditis [162, 163, 164, 165, 166, 167, 168, 169, 170], have been developed: an AlexaFluor680-Prothrombin (AF680-ProT) analog for near infrared preclinical imaging and a ^64^Cu-DPTA-Prothrombin (^64^Cu-DPTA-ProT) analog for use in the more clinically-relevant PET-CT modality [171]. Prothrombin (ProT) is captured and activated by the *S. aureus* secreted proteins, staphylocoagulase and von Willebrand binding protein [172, 173, 174]. Staphylocoagulase is secreted into the circulation and also binds through the C-terminal domain to the fibrin deposited in vegetation, and therefore ProT and its analogs, are incorporated into the vegetation on the heart valve. The AF680-ProT analog can be visualized with FMT/CT, while the ^64^Cu-DPTA-ProT analog requires PET/CT for visualization. Both probes exhibited no effect on the host-clotting cascade while localizing to *S. aureus* vegetations present on heart valves and allowing the monitoring of antibiotic therapy in a murine model of IE. The visualization of a causative microorganism within the vegetation with modified antibiotics has been accomplished by two specific methods: by conjugating vancomycin, an antibiotic which forms hydrogen bonds with the D-alanine moieties present in the Gram-positive cell wall to a fluorochrome, resulting in a probe termed vanco-CW800 for fluorescence imaging; the generation of ^3^H-spiramycin, a macrolide antibiotic that inhibits protein synthesis of the bacterium within the vegetation, providing dual diagnostic and therapeutic effect. In addition, Lee et.al has developed a miniaturized diagnostic magnetic resonance (DMR) system, containing magnetic nanoparticles conjugated to Vancomycin (CLIO-Vanco sensors), that is able to detect Gram positive bacteria in a small volume of unprocessed sample (10 μL) [103, 175, 176]. A recently developed probe for the detection of Enterococci within IE utilizes ^64^Cu-DOTA-anti pili monoclonal antibody for detection of Entercocci in a model of rat endocarditis by PET/CT. The probe, termed MAb 69, is specific for EbpC region of pili. Pili are implicated in biofilm formation and initiation of endocarditis; therefore, the addition of MAb 69 significantly attenuates the pathogenicity of the Enterococci, coupled with high-density labeling of Enterococci *in vivo* [177]. Those probes described here that have been developed for clinical imaging modalities (i.e MRI, SPECT, and PET) achieve the high specificity necessary for proper diagnosis of IE, but currently have not been translated to clinical application.

## 4. Conclusions

Molecular imaging advancements in technology and targeting have revolutionized pre-clinical discoveries and led to clinical advancements in patient diagnostics. All major disease types have benefited from this revolution or more appropriately evolution of the imaging arts including development of new ways to monitor the pathogenesis of cancer and chronic inflammatory diseases, such as atherosclerosis, diabetes, and autoimmune disorders; application of such advancements to infectious disease would lend an increased specificity to diagnosis that would greatly benefit treatment. Currently, the clinical application of molecular imaging to infection is limited to indirect measurement of enhanced localization and metabolic activity of leukocytes via radiotracers for PET or SPECT modalities. The development of diagnostics targeted to the pathogen or disease state would allow the non-invasive identification of the causative microorganism and monitoring of antibiotic therapy for early recognition and eradication of infection. Recently developed diagnostics that satisfy this aim are summarized in Figure 2; ^99m^Tc-labeled-Ciprofloxacin (a fluoroquinolone antibiotic) intercalates into bacterial DNA, and as a pathogen targeted detection method demonstrated initial clinical success; however, variability in discrimination between infection and inflammation initiated its removal from market consideration. The synthetic antimicrobial peptide, UBI 29-41, has demonstrated promising specificity in discriminating infection from inflammation in early clinical trials [105, 107, 108, 109, 113, 114, 115]. However, the continued paucity of FDA approved infection specific agents highlights the difficulty of translating pre-clinical to clinical application due to surmounting the barriers of cost, toxicity, and off-target labeling. Therefore, the application of FDA approved materials in a manner specific to the disease state or pathogen will aid in bypassing the barriers of clinical translation in order to expedite the process of developing infectious disease specific probes. This accelerated method of development is exemplified by the application of USPIO to the diagnosis of vertebral osteomyelitis; it was observed by Bierry *et al*. that two different populations of macrophages infiltrated vertebral osteomyelitis than are found in sterile spinal bone marrow and that injection of USPIO resulted in macrophage uptake and infiltration specific to vertebral osteomyelitis [37]. Targeted methods for specific pathogens would be the most useful for clinical application; however, they are difficult to develop and translate, therefore the novel combination of modalities or probes that are FDA approved may provide a straightforward path for the development of new infectious disease detection agents.

## Figures and Tables

**Figure 1:**
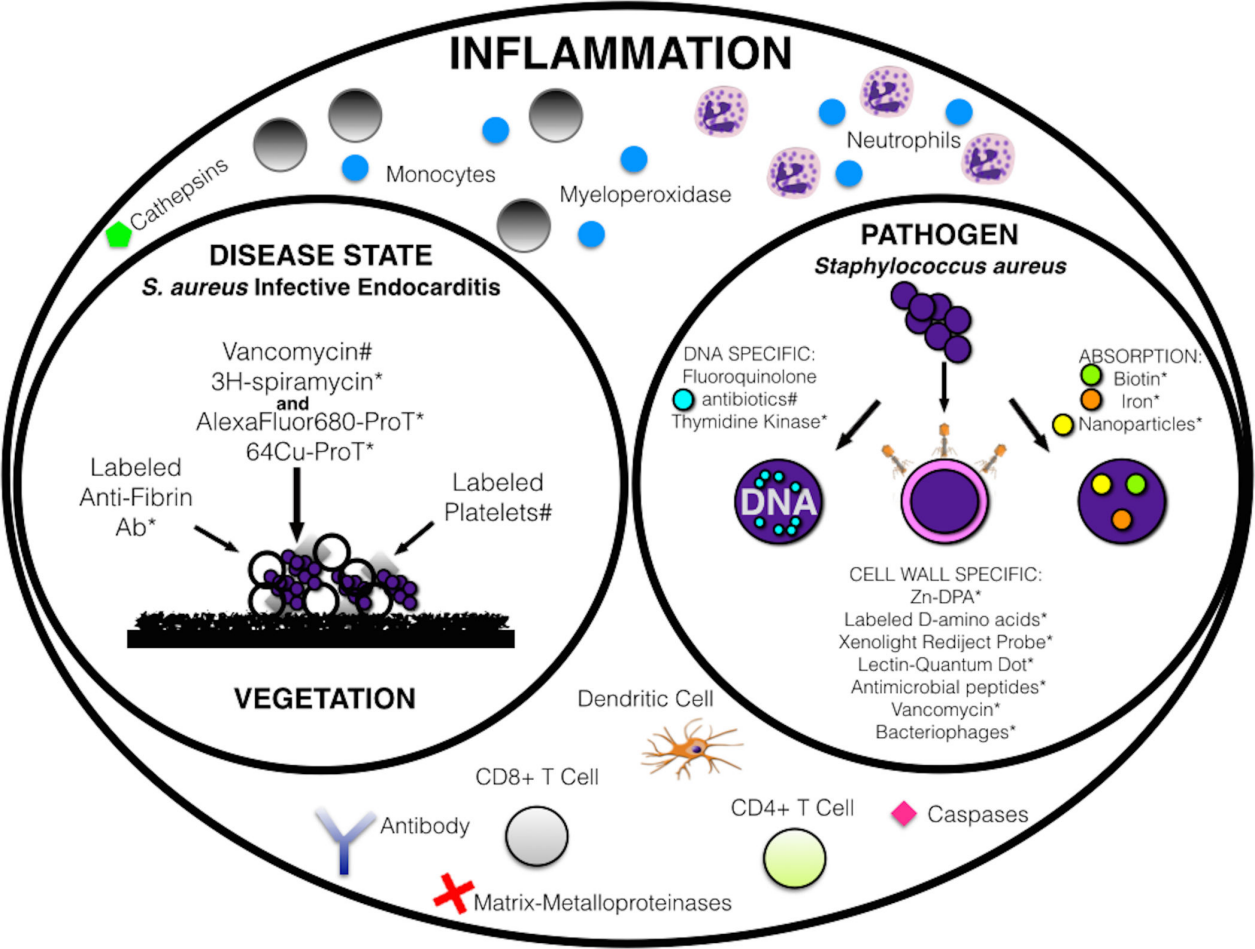
(**a**) Illustrated within each circle “Inflammation,” “Disease State,” and “Pathogen” are markers that can be utilized as molecular targets in the diagnosis of infection. The identification of general inflammatory markers, depicted in the “Inflammation” circle, indicate the inflammation surrounding an infection, but increased specificity of diagnosis can be gained by focusing on targets associated with a disease state or pathogen. Endocarditis and *Staphylococcus aureus* are depicted here as representative of a disease state and associated pathogen of interest, respectively, with current pre-clinical (*) or clinical (#) applications noted.

**Table 1:** 

Labeling Agent	Modality	Half-Life	Pre-Clinical	Clinical (FDA approved)
^99^Technetium	SPECT	6 hours	✓	✓
^89^Zirconium	PET	3.3 days	✓	✓
^67 or 68^Gallium salts	PET, SPECT	3.26 days; 68 minutes	✓	✓
^111^Indium	SPECT	2.8 days	✓	✓
^64^Copper	PET	12.7 hours	✓	✓
^18^Fluorine	PET	109.8 minutes	✓	✓
^123, 124, 125,131^Iodine	PET, SPECT	13.3 hours; 4.18 days; 59.4 days; 8 days	✓	✓
Superparamagnetic iron oxide nanoparticles (SPIO)	MRI	NA	✓	✓
Cross linked iron oxide nanoparticles (CLIO)	MRI	NA	✓	✓
Monocrystalline iron oxide nanoparticles (MION)	MRI	NA	✓	✓
Gadolinium-Chelator (DOTA, DPTA, etc.)	MRI	1.5 hours	✓	✓
Colloidal Quantum Dots	Optical	NA	✓	×
Near Infrared Fluorophore (NIRF) (Indocyanine green)	Optical	150-180 seconds (blood)	✓	✓
NIRF (Cyanine5,5.5,7; AlexaFluor dyes)	Optical	NA	✓	×
Bioluminescence	Optical	NA	✓	×

**Table 2:** 

Clinical Tracer	Imaging Modality	Sample Size	Sensitivity	Specificity	Positive and negative predictive values	Evaluated and Recommended for Endocarditis
**^18^F-FDG**	PET/CT	72	39%	93%	64% 82%	×
**^99m^Tc-HMPAO-WBC**	SPECT/CT	51	90%	100%	100% 94%	✓
**^99m^Tc-anti-NCA-95**	Scintigraphy SPECT	72	79%	82%	×	✓
**^111^In-DPTA-anti-Fibrin mAb**	Scintigraphy	86	97%	72%	×	N/A
**^99m^-Tc-DTPA-anti-Fibrin mAb**	Scintigraphy	94	84.2%	97.6%	×	N/A

Pre-clinical Probe: Host based	Imaging Modality	Pre-clinical Probe: Pathogen based	Imaging Modality	Pre-clinical Probe: Antibiotic based	Imaging Modality
**^99m^Tc-Stannous Pyrophosphate**	Scintigraphy	**Bis-dipicolylamine-Zinc(II)-Carbocyanine**	Optical	**Vancomycin-800CW**	Optical
**^99m^Tc-Annexin V**	Scintigraphy	**AF680-ProThrombin**	FMT/CT	**^3^H-Spiramycin**	Auto-radiography
**^111^In-Platelets**	Scintigraphy	**^64^Cu-DPTA-ProThrombin**	PET/CT		
**Gd-DPTA-anti-Fibrin monoclonal antibody**	MRI	**^64^Cu-DOTA-anti-pilin monoclonal antibody**	PET/CT		
